# Prevention of Pregnancy Loss: Combining Progestogen Treatment and Psychological Support

**DOI:** 10.3390/jcm12051827

**Published:** 2023-02-24

**Authors:** Nana Tetruashvili, Alice Domar, Asher Bashiri

**Affiliations:** 1 V.I. Kulakov Obstetrics, National Medical Research Center for Obstetrics, Gynecology and Perinatology, Ministry of Healthcare of the Russian Federation, 117977 Moscow, Russia; 2 Inception Fertility, Houston, TX 77081, USA; 3 Obstetrics, Gynecology and Reproductive Biology, Harvard Medical School, Boston, MA 02115, USA; 4 Faculty of Health Science, Ben-Gurion University of the Negev, Be’er-Sheva 84101, Israel; 5 Maternity C Ward & Recurrent Pregnancy Loss Prevention Clinic, Maternal Fetal Medicine and Ultrasound, Soroka University Medical Center, Be’er-Sheva 84101, Israel

**Keywords:** dydrogesterone, pregnancy loss, progesterone, progestogen, psychological support

## Abstract

Pregnancy loss can be defined as a loss before either 20 or 24 weeks of gestation (based on the first day of the last menstrual period) or the loss of an embryo or fetus less than 400 g in weight if the gestation age is unknown. Approximately 23 million pregnancy losses occur worldwide every year, equating to 15–20% of all clinically recognized pregnancies. A pregnancy loss is usually associated with physical consequences, such as early pregnancy bleeding ranging in severity from spotting to hemorrhage. However, it can also be associated with profound psychological distress, which can be felt by both partners and may include feelings of denial, shock, anxiety, depression, post-traumatic stress disorder, and suicide. Progesterone plays a key part in the maintenance of a pregnancy, and progesterone supplementation has been assessed as a preventative measure in patients at increased risk of experiencing a pregnancy loss. The primary objective of this piece is to assess the evidence for various progestogen formulations in the treatment of threatened and recurrent pregnancy loss, postulating that an optimal treatment plan would preferably include a validated psychological support tool as an adjunct to appropriate pharmacological treatment.

## 1. Introduction

Pregnancy loss (PL) is defined as either a loss before 20 or 24 weeks of gestation (based on the first day of the last menstrual period) or the loss of an embryo or fetus less than 400 g in weight if the gestation age is unknown [1,2,3,4]. Vaginal bleeding during the first 20 weeks of pregnancy, with or without pain, with a viable, intrauterine fetus and no cervical dilation or effacement, is known as threatened PL [2,3,5,6]. Recurrent PL is defined as either the demise of two or more or three or more pregnancies before the fetus reaches viability [2,7,8,9,10,11]. Approximately 23 million PLs occur worldwide every year, equating to 15–20% of all clinically recognized pregnancies, with 1–2% of women trying to conceive experiencing recurrent losses, although the true incidence may be much higher [1,2,3,12,13].

Both physical and psychological consequences are associated with PL. Early pregnancy bleeding is typical and can range in severity from spotting to hemorrhage [1,2,3,5,13]. The psychological impact can be felt by both partners and may include feelings of denial, shock, anxiety, depression, post-traumatic stress disorder, and suicide [2,13,14,15,16,17]. The consequences of PL are more extensive when considering the increased risk of obstetric complications such as stillbirth in future pregnancies, potential long-term health problems such as cardiovascular disease and venous thromboembolism, and the economic cost to healthcare systems and society: In the United Kingdom alone the economic cost of PL is around £471 million per year [13].

The importance of progesterone in early pregnancy and the probability that progesterone deficiency may lead to PL have long been theorized [18,19,20]. Consequently, exogenous progestogen has been widely used to try to counter the deficiency that can lead to infertility and PL.

In this article, we discuss progestogen treatment in PL and the importance of combining it with psychological support.

## 2. Search Strategy and Selection Criteria

We performed a comprehensive literature search of PubMed and Cochrane Library (from database inception to March 2022) for relevant randomized clinical trials (RCTs), systematic reviews, and meta-analyses, using a combination of free-text search terms and MeSH terms and titles related to ‘Abortion, Threatened’, ‘Abortions, Threatened’, ‘Threatened Abortion’, ‘Threatened Abortions’, ‘Pregnancy Complications’, ‘Abortion, Spontaneous’, ‘abortion’, ‘miscarriage’, ‘progesterone’, ‘17-OHPC’, ‘17α-hydroxyprogesterone caproate’, ‘17p’, ‘17-alphahydroxy-progesterone’, and ‘progestogens’. In addition, we manually searched the reference lists of all relevant articles. Only English-language publications were selected.

## 3. Progestogens in Pregnancy

Progesterone is a female sex hormone with multimodal action that is essential for successful implantation and maintenance of pregnancy [1,2,5,21,22,23,24]. Corpus luteum deficiency along with the corresponding progesterone deficiency has been postulated as a potential etiological factor in PL [2,3]. Consequently, several pharmacological interventions, including progestogens, have been evaluated for the treatment of PL with recommendations reflected in most clinical guidelines (Table 1).

Progestogens are steroid hormones that bind to and activate progesterone receptors [3,43,44,45]. Differences in the chemical structure of progestogens, resulting in variations in their receptor-binding selectivity (androgen, glucocorticoid, estrogen, and mineralocorticoid), potency, and bioavailability can lead to various side effects, including potentially harmful effects on the developing fetus, meaning that not all progestogens are suitable for use during pregnancy [44,45,46]. Due to this cross-reactivity with other receptors, the only progestogens approved for use in pregnancy are progesterone, dydrogesterone, and 17α-hydroxyprogesterone caproate [44,47,48]. These are available in a variety of formulations for clinical use, including oral, vaginal, and intramuscular preparations [44,49,50]. For prevention of PL, studies have suggested that vaginally administered progesterone may be more effective than intramuscular injection [51,52], and recent meta-analyses have concluded that oral progestogens may be as effective as progestogens administered intramuscularly or vaginally [1,2,3].

## 4. Use of Progestogens in Pregnancy Loss

Progesterone supplementation has been assessed in women who present with either threatened PL or women with recurrent PL.

### 4.1. Threatened Pregnancy Loss

Many studies have investigated the ability of progestogens to treat threatened PL. Wahabi et al. performed a pairwise meta-analysis on seven trials (involving 696 participants) to investigate the efficacy of progestogens in the treatment of threatened PL, and found that the use of progestogens compared with placebo or no treatment reduces the risk of PL (risk ratio [RR] 0.64, 95% confidence interval [CI] 0.47–0.87) [3]. Similarly, Wang et al. and Li et al. assessed RCTs comparing progestogen with placebo, no treatment, or any other treatment and reported relative risks of 0.64 (95% CI 0.48–0.85) [53] and 0.73 (95% CI 0.59–0.92) [54], respectively. The conclusion by the latter Li et al. study that benefit was only seen with oral progestogens and not with vaginal progesterone [54] was critiqued by Devall et al., who concluded that the supporting studies utilized were at high risk of bias, and that more high-quality, randomized trials are required to further assess the efficacy and safety of oral progestogens [55]. Li et al. subsequently agreed with this assessment and confirmed that this is the reason that their original publication emphasizing their findings about oral progestogens should be interpreted with caution [56]. More recently, a network meta-analysis evaluated the efficacy and safety of different progestogens in the treatment of threatened PL and confirmed that progestogens were effective in reducing the risk of PL [57].

### 4.2. Recurrent Pregnancy Loss

A number of studies, including RCTs, have evaluated the efficacy of progestogens in recurrent PL. Haas et al. performed a pairwise meta-analysis on 10 studies (involving a total of 1684 participants) to investigate the efficacy of progestogens in the treatment of recurrent PL. They compared progestogen with placebo or no treatment and found that the use of progestogens reduced the risk of recurrent PL (RR 0.73, 95% CI 0.54–1.00) [2].

### 4.3. Which Progestogen Should Be Used for Pregnancy Loss?

Much emphasis has been put on the ability of micronized vaginal progesterone (MVP) to reduce the risk of future PL in women suffering from recurrent PL. In the PROMISE study—a double-blind, placebo-controlled trial in which 836 women with unexplained recurrent PL were randomized to receive twice-daily MVP suppositories (*n* = 404) or matching placebo (*n* = 432) from soon after a positive pregnancy test to Week 12 of gestation—MVP treatment did not significantly (*p* = 0.45) improve the live birth rate (LBR) (65.8% MVP versus 63.3% placebo [RR 1.04, 95% CI 0.94–1.15]) [58]. Similarly, the PRISM trial (another double-blind, placebo-controlled, randomized trial evaluating MVP versus placebo in 4153 women with vaginal bleeding early in pregnancy) failed to show a statistically significant difference in the LBRs (primary endpoint) between treatment groups: Of 2079 women receiving MVP and 2074 receiving placebo the LBR was 75% in the MVP group versus 72% in the placebo group (RR 1.03, 95% CI 1.00–1.07; *p* = 0.08) [59]. Prespecified subgroup analysis of the data revealed a significant subgroup effect in women who have experienced ≥3 previous PLs (LBR 72% with MVP versus 57% with placebo: RR 1.28, 95% CI 1.08–1.51; *p* = 0.007) [59]. Further post hoc subgroup analysis suggested that MVP was effective in women who have experienced ≥ 1 previous PL (LBR 75% with MVP versus 70% with placebo; RR 1.09, 95% CI 1.03–1.15; *p* = 0.003) [60].

The PRISM subgroup results support the findings from meta-analyses which concluded that progestogens may prevent PL in women with recurrent PL [2,42,61], leading the National Institute for Health and Care Excellence to revise its guidelines and recommend the off-label use of MVP [26]. However, the benefit of MVP over other forms of progesterone is not clear. A double-blind, randomized, placebo-controlled trial investigating the progestogen dydrogesterone demonstrated efficacy in women with recurrent PL [62], but there are no similarly robust studies for other progestogens.

## 5. Routes of Progestogen Administration and Patient and Physician Preferences

Progestogens differ in their potency, receptor-binding selectivity, bioavailability, and route of administration, and these factors should guide the choice of the most appropriate treatment [44,46]. Despite micronization, oral progesterone is still hindered by poor bioavailability [44], meaning that high doses are required, resulting in side effects such as drowsiness, nausea, and headaches [46,63,64,65]. Vaginal administration of micronized progesterone does improve uterine concentrations but can cause irritation, vaginal discharge, and bleeding; is often uncomfortable; or may be washed out if bleeding is severe [64,66,67,68]. Optimal blood levels are achieved with intramuscular progesterone but can induce abscess formation and be extremely painful [67].

Vaginal and oral preparations are the most widely studied in PL and it is highly likely that patients will have a formulation preference based on convenience and tolerability. While it may be reasonable to assume that the convenience and lack of vaginal side effects may lead to a preference for oral administration, studies in this respect have not been conducted in the recurrent PL setting. However, studies involving other gynecological treatments have shown that both patients and healthcare professionals generally prefer oral administration over vaginal administration [69,70,71,72], and oral dydrogesterone has become widely used during pregnancy.

## 6. Dydrogesterone

**Figure 1:**
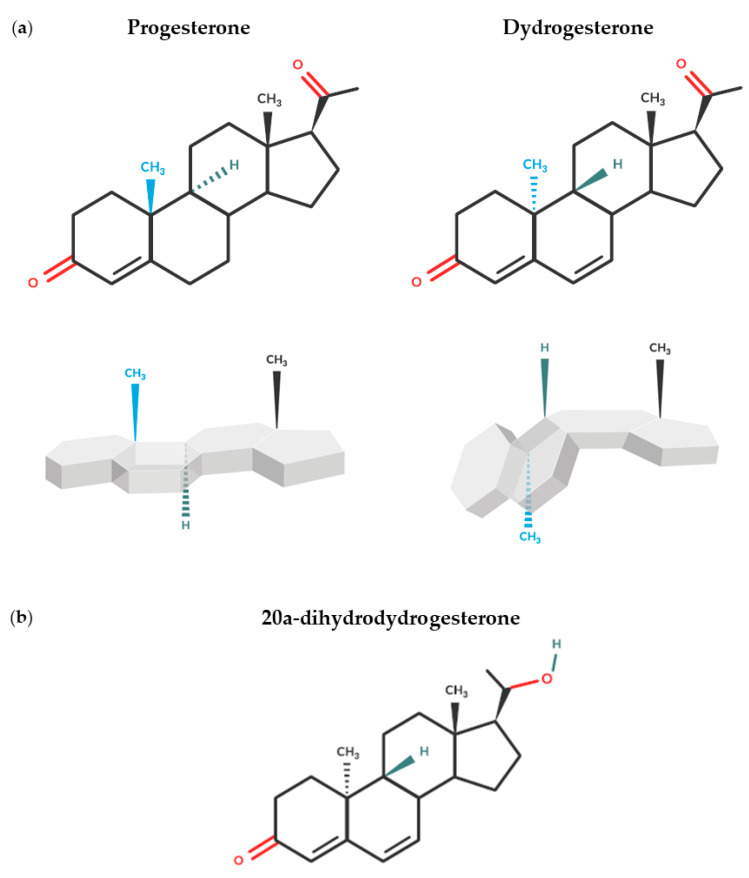
Chemical structures of (**a**) progesterone and dydrogesterone and resulting difference in spatial conformation and (**b**) main dydrogesterone metabolite, 20α-dihydrodydrogesterone.

Dydrogesterone is a retroprogesterone and a potent and selective oral progesterone receptor agonist [44,73]. A change in the spatial orientation of a methyl group at carbon 10 and a double bond between carbons 6 and 7 differentiate dydrogesterone from progesterone and give it a “bent” shape (Figure 1a) [46,64,73]. This unique structure results in high oral bioavailability, while the high selectivity of dydrogesterone for the progesterone receptor means that it can be administered at doses 10–20 times lower than those of oral micronized progesterone [44,64,74]. Furthermore, unlike other forms of progesterone, dydrogesterone‘s main metabolite, 20α-dihydrodydrogesterone (Figure 1b), exhibits similar progestogenic selectivity to the parent molecule, thus minimizing unwanted adverse events (AEs) [44].

### 6.1. Efficacy of Dydrogesterone in Pregnancy Loss

There are publications not relevant for discussion, due to their age, quality, and design [75]; however, more recently, there is a robust body of evidence (including RCTs, reviews, and meta-analyses) to support the efficacy of dydrogesterone in both threatened (Table 2) and recurrent (Table 3) PL.

Three RCTs have shown that women with threatened PL receiving dydrogesterone had significantly (*p* < 0.05) lower PL rates compared with those receiving bed rest with or without supportive care [76,77,78]. This has been supported by various meta-analyses showing a significant (*p* = 0.001) reduction in the rate of PL with oral progestogens, including dydrogesterone [3,6]. In each case, the comparators had little or no treatment effect and did not significantly reduce PL (Table 2). In a more recent meta-analysis, Zhao et al. assessed the efficacy and safety of various progestogens across 59 RCTs and a total of 10,424 women and also concluded that oral dydrogesterone was more effective in the treatment of threatened PL than vaginal progesterone [57].

Key efficacy data for dydrogesterone in recurrent PL is summarized in Table 3. Two RCTs have demonstrated that women with recurrent PL receiving dydrogesterone had significantly (*p* < 0.05) lower PL rates than those receiving placebo or no additional treatment [62,81]. Two meta-analyses reported similar beneficial effects with dydrogesterone in women with recurrent PL. One assessed three studies (*n* = 509) and concluded that dydrogesterone caused a substantial reduction in the rate of PL versus placebo or conservative treatment [82]. Another, which assessed a total of 10 studies (*n* = 1684) looking at progestogens versus placebo or no treatment [2], concluded that there was a numerical decrease in the PL rate with progestogen treatment, including in studies of dydrogesterone. It is noteworthy that, in this last meta-analysis, two studies showing a clear reduction in the risk of PL with progestogen treatment were two of the three that used dydrogesterone [2,62,81]. These results are supported by another RCT investigating dydrogesterone versus vaginal progesterone in women with recurrent PL, which found that less time was required for complete cessation of bleeding in patients treated with dydrogesterone versus those treated with vaginal progesterone (53.90 ± 9.09 versus 94.60 ± 7.29 h, *p* < 0.0001) [83].

Recently, Devall et al. carried out a network meta-analysis (which pooled direct and indirect evidence on relative treatment effects to achieve a single coherent analysis) to assess progestogens for the prevention of PL and concluded that progestogens probably make little or no difference in LBR for women with threatened or recurrent PL; however, MVP may increase the LBR for women with a history of ≥ 1 previous PLs and early pregnancy bleeding [1]. They also concluded that there is still uncertainty over the effectiveness and safety of alternative progestogen treatments for threatened and recurrent PL [1].

It is interesting to note that the network meta-analysis carried out by Devall et al. included seven RCTs, only one of which was a dydrogesterone-related publication [1]. In contrast, Zhao et al. included a much larger number of RCTs, with a large proportion of those investigating dydrogesterone [57]. In fact, current guidelines reflect the body of evidence and support the use of dydrogesterone in both threatened and recurrent PL, reflecting a good efficacy and safety profile, with a low incidence of maternal and fetal complications (Table 1). The subjective nature of the decision to exclude or include studies in network meta-analyses re-enforces the value and importance of always considering the primary data and conducting systematic reviews with meta-analyses.

### 6.2. Safety of Dydrogesterone in Pregnancy

Dydrogesterone has been marketed and used since the 1960s for a number of conditions associated with progesterone insufficiency and is indicated for the treatment of both threatened and recurrent PL in numerous countries worldwide [47,84]. Based on dydrogesterone cumulative exposure data from April 1960 to April 2021, it is estimated that the post-marketing patient exposure is 137.5 million patient treatment years. In 2014, based on sales figures, it was reported that an estimated >20 million pregnancies were exposed to dydrogesterone in utero from April 1960 to April 2014 [85]. Between 1977 and 2005, pharmacovigilance data has identified only 28 cases of congenital defects with a potential link to fetal dydrogesterone exposure [86]. This may be due to the unique structure of dydrogesterone, which allows efficacy with oral administration at low therapeutic doses, avoiding the tolerability issues associated with vaginal administration of progesterone. In addition, dydrogesterone’s high selectivity for progesterone receptors may help to limit the risk of side effects [44], as supported by dydrogesterone’s well-established safety profile, which reflects no notable safety concerns for the mother or the developing fetus when used during pregnancy [87].

#### 6.2.1. Maternal Safety

Dydrogesterone has been shown to have a good safety profile with a low incidence of maternal complications. It also seems to be as well tolerated as vaginal progesterone in safety analyses from studies assessing dydrogesterone’s use in luteal phase support during in vitro fertilization (IVF) as well as those investigating dydrogesterone’s use in threatened and recurrent PL [74].

**Figure 2:**
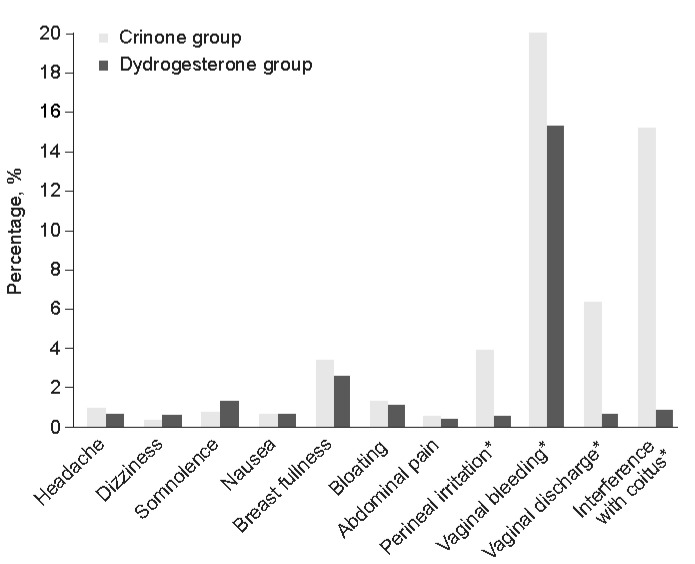
Tolerability of dydrogesterone versus vaginal progesterone gel (8%) in luteal phase support [68]. * *p* < 0.05 by Chi-square test, in favor of Crinone group. Reprinted from Eur J Obstet Gynecol Reprod Bio, Vol 186, Tomic V, Tomic J, Klaic DZ, et al. Oral dydrogesterone versus vaginal progesterone gel in the luteal phase support: randomized controlled trial, Pages 49–53, Copyright 2015, with permission from Elsevier.

An RCT comparing dydrogesterone versus vaginal progesterone gel in luteal phase support confirmed that patients receiving progesterone gel experienced a higher incidence of vaginal irritation and discharge, vaginal bleeding, and interference with sexual activity compared with patients receiving dydrogesterone (Figure 2) [68]. A meta-analysis of the individual participant data from the Lotus I and II luteal phase support trials confirmed that the incidence of maternal AEs was similar between the dydrogesterone and MVP treatment groups, with the most common AE being vaginal bleeding [88,89,90]. These results are supported by a more recent, retrospective cohort study, which investigated whether the use of dydrogesterone versus MVP affected pregnancy outcomes in frozen embryo transfer and found that maternal complications were similar in both patient groups [91].

This favorable maternal safety profile has been mimicked in studies investigating dydrogesterone’s use in both threatened and recurrent PL, which have reported no significant differences overall in maternal complications with dydrogesterone versus oral micronized progesterone or placebo [79,80].

#### 6.2.2. Fetal Safety

A favorable safety profile has also been noted for dydrogesterone when considering fetal complications such as low birth weight, neonatal death, and congenital anomalies [2,74].

In the Lotus I and II trials, as well as subsequent meta- and subpopulation analyses, the incidence of congenital, familial, and genetic disorders was low and similar between oral dydrogesterone and MVP gel [74,84,88,89,90,92]. These results have been supported by more recent retrospective cohort studies. One such study assessed 3556 infants in China after IVF using a dydrogesterone + human menopausal gonadotropin protocol (*n* = 1429) or gonadotropin-releasing hormone-agonist short protocol (*n* = 2127) and found that the two protocols showed no differences in birth weight characteristics and had a similar incidence of congenital malformations following exposure to dydrogesterone [93]. Another, more recent, retrospective cohort study investigated whether the use of dydrogesterone versus MVP affected pregnancy outcomes in frozen embryo transfer and found that no fetal anomalies were observed in either treatment group [91] Similarly, studies of threatened and recurrent PL have also reported no significant differences in fetal/neonatal complications with dydrogesterone versus placebo or MVP [74].

There have been publications claiming that dydrogesterone has teratogenic effects. Due to poor design and lack of adherence to the basic principles of epidemiological research (differences in the maternal population leading to confounding, lack of confirmed dydrogesterone exposure, pooling of different heart defects during assessment, and disregarding comorbidities and socioeconomic status), a causal relationship between dydrogesterone and heart defects cannot and should not be inferred from the study conducted by Zaqout et al. [94]. Similarly, another study published in 2020 claimed that dydrogesterone confers teratogenic effects after exposure to the recommended doses in pregnant women [95]; however, substantial concerns regarding the study design, statistical analysis, inconsistencies and inaccuracies of data reporting, and validity of the conclusions have since prompted the journal to retract the article.

Based on its extensive use, a substantial teratogenic risk of dydrogesterone with no safety issues seems very unlikely [96]. The validity and continued use of retracted and poor-quality data was recently questioned by Katalinic et al., who performed a meta-analysis of six RCTs and concluded that use of dydrogesterone during the first trimester of pregnancy was not associated with a significant increase in risk of congenital abnormalities (RR 0.96, 95% CI 0.57–1.62). Moreover, they support the use of dydrogesterone, if indicated, in the treatment of threatened or recurrent PL [87].

## 7. Complementing Pharmacological Treatment with Psychological Support

### 7.1. The Psychological Impact of High Risk Pregnancies on Women and Pregnancy Outcomes

A pregnancy is considered high-risk when there is the increased probability of an adverse outcome for either the mother or the fetus that can occur due to a variety of reasons, such as: the development of hypertensive disorders of pregnancy, gestational diabetes mellitus, changes in the cervix and placental abruption, and/or serious abnormalities that occur in the baby [97,98,99].

It is well documented that high-risk pregnancies can have a negative impact on both the psychological and physical wellbeing of women, causing negative emotions such as fear, shock, grief, guilt and distress [97,100,101]. There is also increasing evidence which suggests that psychological wellbeing may affect pregnancy outcomes. For example, elevated levels of distress during high-risk pregnancy may be associated with detrimental effects on the baby such as low birth weight and preterm delivery, and recent publications generally conclude that psychological interventions which can increase a sense of control could help improve pregnancy outcomes [97,99,101].

While more emphasis is now being placed on the need to explore the emotional and psychological challenges as well as the medical aspects during high-risk pregnancies, the aim of this paper is to focus on those studies that have assessed the psychological wellbeing of women who are at high risk of suffering a PL.

### 7.2. The Psychological Impact of Pregnancy Loss

Profound emotional distress and psychological morbidity is commonly experienced by women who present with bleeding in threatened PL as well as women with recurrent PL [1,2,3,102,103]. However, a difference between the psychological impact of a single loss and multiple PLs has been reported, where depressive symptoms or the risk of psychological distress were found to increase with the number of prior losses [104]. Indeed, multiple studies have reported high levels of anxiety and depression in women experiencing recurrent PLs, with the repetitive nature of the loss adding to the emotional impact and the early stages of a new pregnancy representing a particularly challenging time for women due to anxiety over the possibility that they will experience a further loss [9,14,15,17,102,103,105]. This is highlighted by the results of a focus group study which reported that women who had experienced recurrent losses were unable to confidently feel hope or joy during the waiting period and often used ‘bracing for the worst’ as a coping strategy during this time [106].

Rather than repeatedly endure this period of uncertainty, and with inadequate emotional support and/or coping skills, some women who have experienced recurrent PLs decide not to attempt conception again [16,107], emphasizing the need to combine pharmacological treatments for threatened and recurrent PL with some form of psychological support or counselling for both the women and their partners during this crucial period [108,109,110].

### 7.3. How Does a Woman’s Psychological State Affect the Risk of Pregnancy Loss?

Multiple studies have looked at the effect of stress on the risk of PL. A large prospective study of work-related stress in nearly 4000 pregnant women reported that stress was associated with a higher risk of PL in women over 32 years of age (*p* = 0.04), women who smoked (*p* = 0.02), and in first pregnancies (*p* = 0.06) [111]. Similarly, a cohort study of 1098 pregnant women reported that higher levels of perceived stress were associated with subsequent PL (*p* = 0.024) [112]. In support of these data, a meta-analysis of eight case–control and cohort studies concluded that psychological stress was associated with an increased risk of PL (odds ratio 1.42) [113], and, more recently, these results were corroborated by a prospective cohort study of 293 women attending an early pregnancy assessment unit which reported that lack of emotional wellbeing was associated with an increased risk of PL [114].

### 7.4. Can Psychological Support Help Improve the Wellbeing of the Patient and Their Partners?

It has been previously suggested that there is a lack of evidence to support counselling following early PL [115]. Nevertheless, more recently it has been reported that women and their partners have expressed an unmet need for psychological interventions that will provide emotional support and strategies to help them cope in the period during and after a PL, during a subsequent pregnancy, and when deciding whether to try to conceive again [105,116,117]. Despite the known psychological and emotional effects of PL, and the potential impact of stress on pregnancy outcomes, limited support and counselling is available during the early stages of a new pregnancy [9]; however, encouragingly, the field has begun to slowly evolve in recent years to focus more keenly on the patient experience, with more studies now reporting the opinions and feelings of not only women but also their partners, and confirming that both women and their partners are at risk of developing anxiety and depression [118,119,120,121,122].

While psychological and supportive care following a PL has not been extensively studied, a number of psychological interventions, such as counselling, mindfulness-based psychotherapy, cognitive behavioral therapy, and positive reappraisal coping intervention (PRCI), have been investigated in the setting of recurrent PL and yielded promising results; preliminary evidence of benefits in terms of stress, depression, and anxiety has been reported, with the PRCI notably receiving positive feedback from patients in terms of ease of use and effects on mindset [9,107,123,124,125,126]. Affirmation of the effectiveness of these interventions has been provided by a recent study of 294 women in which 72.7% (*n* = 176) went on to achieve a live birth, and in which supportive care was reported to probably be the single most effective therapy [127]. Another study in women who have experienced recurrent PL confirmed that patient-centered care may have a significant impact on the chance of a live birth in the next pregnancy; in general, the LBR for these couples was around 80% [103]. More recent studies have confirmed that health professionals also recognize the need for better care following a PL and are aware that urgent action is needed to improve the options they provide, including increased information about PL, more emotionally sensitive care at the time of PL, and more psychological support options [108,109,110,128]. Crucially, and regardless of intervention, the majority of guidelines emphasize this unmet need and the important role of supportive care [7,8,11].

## 8. Future Research and Conclusions

We have highlighted that progestogens such as dydrogesterone, could be beneficial in the treatment of threatened and recurrent PL. Studies are ongoing to further our understanding around patient-related factors, such as the diversity of the reproductive microbiome and its relation to pregnancy outcomes [129,130,131], as well as treatment-related factors, such as: how combination treatment for intrauterine abnormalities, immune status, and thyroid function—referred to as the ‘OPTIMUM’ (OPtimization of Thyroid function, IMmunity, and Uterine Milieu) treatment strategy—might potentially improve pregnancy outcomes [132]; the optimal duration of progestogen treatment; the impact of beginning progestogen treatment prior to conception and continuing into pregnancy (a practice which is already used in Russia and which is recommended in the national clinical guidelines) [39]; and the genetic diversity of the progesterone receptor and the specific patient populations that could derive the greatest benefit from progestogen supplementation in pregnancy [133,134,135]. Another consideration is the potential relationship exogenous progestogens may have in mood-related adverse events, e.g., anxiety and depressive symptoms. Research around the use of hormonal contraception has acknowledged that there is a subset of women who suffer with mood-related side effects, despite the introduction of lower dose pills and alternative delivery methods [136,137]. More recently, hormonal contraceptive use has been associated with increased depression and impairment of emotion recognition, with the suggestion that the progestogen in the hormonal contraception is what causes mood problems [138,139]. This, combined with some evidence suggesting a link between endogenous progesterone levels and anxiety [140] as well as a role for allopregnanolone (a 3-alpha reduced metabolite of progesterone) dysregulation in mood changes [139,141], means that there may be cause for concern regarding the use of progestogens for PL, especially as patients are already vulnerable to increased levels of anxiety and depression. However, continued research into the etiology of hormonally induced mood symptoms remains inconclusive, with few studies able to directly connect levels of hormones to psychopathology [137,139]; much more research is needed to be able to identify populations of women who might be at greater risk of mood-related adverse events following treatment with progestogens. Indeed, a more in-depth appreciation of all of these factors could increase our understanding of the progestogen mechanism of action and help optimize and personalize future patient treatment.

Given the emotional trauma many women experience following a PL, we have also postulated that structured psychological support is crucial to safeguard the wellbeing of the patient to reassure her that she is doing everything possible to support her pregnancy, help her and her partner cope with the emotional impact of PL, and support them through the stressful waiting period in subsequent pregnancies. Further research is needed to determine the most effective form of psychological intervention for these patients, with recommendations for implementation. As we try to elucidate how findings from novel research may impact treatment outcomes, it would be prudent to consider a reconceptualized, holistic approach to the treatment of threatened and recurrent PL in order to provide optimal care and support for women and their partners. While novel studies are needed to confirm whether a combined pharmacological and psychological intervention is superior to either approach alone, a reimagined treatment plan would preferably combine a validated psychological support tool as an adjunct to appropriate pharmacological treatment. With a keen focus on the patient’s wishes, this plan would also aim to address some of the diverse aspects that may contribute to PL, thus maximizing future chances of a live birth.

## Figures and Tables

**Table 1:** Summary of guidelines for treatment of pregnancy loss.

**Guidelines for Threatened PL**	
**International and Regional Guidelines/Citation**	**Recommendations/Statements**
EPC guideline (2015 [5])	For women presenting with a clinical diagnosis of threatened PL, there is a reduction in the rate of spontaneous PL with the use of dydrogesterone
Australia and New Zealand, RANZCOG guidelines [25]	Progestogen supplementation until the second trimester in women presenting with a clinical diagnosis of threatened miscarriage may reduce the rate of spontaneous miscarriage and may be considered
**National Guideline/Citation**	**Recommendations/Statements**
UK, NICE guidelines [26]	Offer vaginal micronized progesterone 400 mg BID to women with an intrauterine pregnancy confirmed by a scan if they have vaginal bleeding and have previously had a miscarriage
Saudi Arabian guidelines [27]	Oral progestogens, namely dydrogesterone, are well tolerated and effectively reduce miscarriages in women at risk of threatened miscarriage
	Available evidence is insufficient to recommend the use of vaginal progestogens ^a^ for the treatment of threatened miscarriage
Russian clinical guidelines (miscarriage) [28]	Dydrogesterone or micronized progesterone should be prescribed to women with threatened miscarriage as a pregnancy-saving therapy. Both dydrogesterone and micronized progesterone have good safety profiles
Malaysian guidelines [29]	Women may be treated with: Oral dydrogesterone (10 mg BID), from the onset of bleeding until 1 week after bleeding has stopped Oral dydrogesterone (10 mg BID from the onset of bleeding until the 16th week of pregnancy), or micronized progesterone (400 mg BID vaginal/rectal administration) if the woman has a history of ≥1 previous miscarriage
FOGSI guidelines [30]	Women may be treated with MVP (400 mg/day until bleeding stops) or oral dydrogesterone (40 mg loading dose followed by 20–30 mg/day until 7 days after bleeding stops)
Chinese guidelines [31]	Oral dydrogesterone is the first choice—40 mg immediately, followed by 10 mg every 8 h until symptoms abate; then continue oral dydrogesterone for 1 to 2 weeks
Vietnamese guidelines [32]	Endocrine medication, such as progesterone 25 mg × 2 ampoules (intramuscular injection)/day, if there is evidence of endocrine insufficiency or to relax the uterine muscles
Vietnamese Hung Vuong Hospital guidelines [33]	Treat symptoms after excluding infectious threatened miscarriage: Progesterone 25 mg/ampoule (intramuscular injection); 1–4 ampoules/day Semi-synthetic (dydrogesterone), maximum dose 40 mg/day; Oral progesterone, maximum dose 600 mg/day
Taiwanese guidelines, Taiwan Society of Perinatology 2022 [34]	Oral dydrogesterone is the **only** recommended medicine: 40 mg immediately followed by 10 mg BID until symptoms are in complete remission; then continue dydrogesterone 10 mg BID for 1 to 2 weeks
Indonesian guidelines [35]	Natural progestogens can be used as therapy for threatened miscarriage Recommendations for treatment include: Dydrogesterone initial dose of 40 mg orally followed by 3 × 10 mg until bleeding stops. Then taper off 2 × 10 mg up to 16 weeks gestation Progesterone 2 × 400 mg orally until 12 weeks gestation Pessary progesterone 2 × 400 mg rectally Progesterone gel 8%, 90 mg, 1–2 times/day vaginally Hydroxyprogesterone 250 mg/week, intramuscularly
Mexican guidelines [36]	Current evidence is insufficient for prescribing progesterone; however, the use of progesterone is recommended for avoiding an emergency and unnecessary medical procedure, and for reassuring the patient
Philippines [37]	Data are limited and further investigation is required; however, there is some evidence that progesterone treatment may reduce the risk of a PL even in women without a history of recurrent PL Progesterone is available for use in three forms: oral, intramuscular, and intravaginal. Among these preparations, oral progesterone is more effective for the treatment of women with threatened PL Progesterone, especially oral dydrogesterone, may be given to treat threatened PL in women with a history of recurrent PL
**Guidelines for Recurrent PL**	
**International and Regional Guidelines/Citation**	**Recommendations/Statements**
EPC guideline [5]	For women presenting with a clinical diagnosis of recurrent miscarriage (three or more), there is a reduction in the rate of miscarriage with the use of dydrogesterone
ESHRE guideline [7]	Vaginal progesterone during early pregnancy has no beneficial effect in women with unexplained recurrent PL. There is some evidence that dydrogesterone, initiated when fetal heart action can be confirmed, may be effective, but more trials are needed
German (DGGG), Austrian (OEGGG), and Swiss (SGGG) Societies of Gynecology and Obstetrics guideline [10]	Synthetic progestogens ^b^ can be administered to women with idiopathic recurrent miscarriage in the first trimester of pregnancy to prevent miscarriage
	Treatment with natural micronized progesterone in the first trimester of pregnancy to prevent miscarriage is not recommended for women with idiopathic recurrent miscarriage
**National Guideline/Citation**	**Recommendations/Statements**
UK, Royal College of Obstetricians and Gynaecologists guidelines [11]	There is insufficient evidence to evaluate the effect of progesterone supplementation in pregnancy to prevent a miscarriage in women with recurrent miscarriage
American Society for Reproductive Medicine [8]	In patients with three or more consecutive miscarriages immediately preceding their current pregnancy, empiric progestogen administration may be of some benefit
Saudi Arabian guidelines [27]	Oral progestogens, namely dydrogesterone, are well tolerated and effectively reduce miscarriages in women at risk of idiopathic recurrent miscarriage
	Available evidence is insufficient to recommend the use of vaginal progestogens ^a^ for the treatment of recurrent miscarriage
Israeli guidelines [38]	Progesterone support has been found to provide an advantage in women with recurrent PLs. Meta-analyses and systematic reviews have found an advantage in specific preparations such as dydrogesterone
Russian clinical guidelines (recurrent miscarriage) [39]	Dydrogesterone or micronized progesterone should be prescribed to women with recurrent miscarriage before pregnancy in luteal phase or from the first visit during pregnancy until 20 weeks of pregnancy. Both dydrogesterone and micronized progesterone have good safety profiles
Russian clinical guidelines (normal pregnancy) [40]	Oral dydrogesterone (20 mg/day) or micronized progesterone (200–600 mg/day, oral or vaginal) should be prescribed to women with recurrent miscarriage from the first visit until 20 weeks of pregnancy
Malaysian guidelines [29]	Can consider progesterone therapy in women with unexplained recurrent miscarriages: There is some evidence that oral dydrogesterone is effective if initiated when fetal heart activity is confirmed
FOGSI guidelines [30]	Women may be treated with oral dydrogesterone (10 mg BID until 20 weeks of pregnancy) or MVP (400 mg/day until 20 weeks of pregnancy)
Chinese guidelines [31]	Oral dydrogesterone is the first choice: 30 mg/day
Taiwanese guidelines, Taiwan Society of Perinatology 2022 [34]	Oral dydrogesterone 10 mg BID or MVP 400 mg BID is to be given when pregnancy is confirmed. Treatment should be continued until 20th week of gestation
Indonesian guidelines [41]	Administration of dydrogesterone significantly reduces the chance of recurrent miscarriage and increases pregnancy rate (Recommendation A) Administration of dydrogesterone is more effective and beneficial when started as soon as fetal heart activity is confirmed/from the luteal phase, because it has been shown to reduce the risk of miscarriage (Recommendation A)
Vietnamese guidelines [32]	Treatment option is based on the cause of recurrent miscarriage; in the case of endocrine insufficiency: endocrine supplements such as progesterone 25 mg × 2 ampoules (deep intramuscular injection)/day, estrogen 2 mg/day
Philippines [37]	There appears to be benefit in giving progestogens orally (medroxyprogesterone acetate 10 mg/day or dydrogesterone 20–30 mg/day until ≥12 weeks) or intramuscularly (hydroxyprogesterone caproate 500 mg/week until 36 weeks) in preventing PL among women who have a history of recurrent PL There is insufficient evidence to show any preferred route, dosage, or duration of treatment

^a^ Capsule, suppository, micronized, or gel; ^b^ supporting dataset included dydrogesterone in the synthetic progestogen group [42]. BID, twice daily; EPC, European Progestin Club; ESHRE, European Society of Human Reproduction and Embryology; FOGSI, Federation of Obstetric and Gynaecological Societies of India; MVP, micronized vaginal progesterone; NICE, National Institute for Health and Care Excellence; PL, pregnancy loss; RANZCOG, Royal Australian and New Zealand College of Obstetricians and Gynaecologists; UK, United Kingdom.

**Table 2:** Dydrogesterone in threatened pregnancy loss.

			Rate of PL			
RCT	Dydrogesterone Treatment	Control Treatment	Dydrogesterone	Control or Untreated Group	RR/OR (95% CI)	*p* Value
[76]	Dydrogesterone 40 mg, followed by 10 mg BID (*n* = 74) until bleeding stopped, or conservative therapy with bed rest and folic acid	All women received bed rest and folic acid (*n* = 80)	4.1%	13.8%	–	*p* = 0.037
[77]	Standard supportive care and dydrogesterone 10 mg BID (*n* = 86) until 1 week after bleeding stopped	All women received standard supportive care ^a^ (*n* = 60)	17.5%	25.0%	–	*p* < 0.05
[78]	Dydrogesterone 40 mg (stat dose), followed by 10 mg BID (*n* = 96) until Week 16	Conservative management with bed rest only (*n* = 95)	12.5%	28.4%	–	*p* < 0.05
[79]	Dydrogesterone 10 mg BID (*n* = 71) for 2 weeks	Oral micronized progesterone 200 mg BID (*n* = 70) for 2 weeks	15.2%	10.2%	–	*p* = 0.581
[80]	Dydrogesterone 40 mg (stat dose), followed by 10 mg TID (*n* = 203) until Week 12 or 1 week after cessation of bleeding	Placebo (*n* = 203)	12.8%	14.3%	RR: 0.897 (0.548–1.467)	*p* = 0.772
			**Rate of PL**			
**Meta-Analysis**	**Dataset**	**Main Result**	**Dydrogesterone**	**Control**	**RR/OR** (**95% CI**)	***p*** **Value**
[67]	Dydrogesterone vs. placebo or conservative treatment Five studies (*n* = 660)	Significant ^b^ reduction in the miscarriage rate with dydrogesterone	13.0%	24.0%	OR: 0.47 (0.31–0.70)	NA ^b^
[6]	Dydrogesterone vs. control (conservative treatment) Three studies (*n* = 491)	Significant reduction in the miscarriage rate with dydrogesterone	11.7%	22.6%	OR: 0.43 (0.26–0.71)	*p* = 0.001
	Vaginal progesterone vs. control (placebo or conservative treatment) Four studies (*n* = 286)	Decrease in the miscarriage rate with vaginal progesterone (not significant)	15.4%	20.3%	OR: 0.72 (0.39–1.34)	*p* = 0.3
[3]	Oral progestogen vs. no treatment Three studies (*n* = 408); dydrogesterone two studies (*n* = 337)	Significant reduction in the miscarriage rate with oral progestogens (including dydrogesterone)	NA	NA	RR: 0.57 (0.38–0.85)	*p* = 0.0059
	Vaginal progesterone vs. placebo Four studies (*n* = 288)	Little or no treatment effect with vaginal progesterone	NA	NA	RR: 0.75 (0.47–1.21)	*p* = 0.24
[54]	Progestogens vs. placebo or no treatment Ten studies (*n* = 5104); dydrogesterone vs. placebo or no treatment, four studies (*n* = 563)	Oral progestogens may have benefits on rates of PL: benefit only seen with oral progestogen and not with vaginal progesterone	18.5%	21.9%	RR: 0.73 (0.59–0.92)	*p* = 0.01
[57]	Progestogens vs. other progesterone treatment or placebo 59 studies (*n* = 10,424); dydrogesterone vs. other progesterone treatment or placebo, 49 studies (*n* = 2793)	Dydrogesterone significantly reduced the risk of miscarriage vs. vaginal, IM, and oral micronized progesterone or placebo	NA	NA	Vaginal OR: 0.50 (0.34–0.74)	*p* = 0.002
					IM OR: 0.41 (0.32–0.54)	*p* = 0.006
					Oral OR: 0.37 (0.28–0.48)	*p* < 0.001
					Placebo OR: 0.42 (0.29–0.61)	*p* < 0.001

^a^ Iron, folic acid, multivitamin supplements, and recommended bed rest; ^b^*p* value not stated. BID, twice daily; CI, confidence interval; IM, intramuscular; NA, not applicable; OR, odds ratio; PL, pregnancy loss; RCT, randomized clinical trial; RR, risk ratio; TID, three times daily.

**Table 3:** Dydrogesterone in recurrent pregnancy loss.

			Rate of PL			
RCT	Dydrogesterone Treatment	Control Treatment	Dydrogesterone	Control or Untreated Group	RR/OR (95% CI)	*p* Value
[81]	Dydrogesterone 10 mg BID (*n* = 82) until gestation Week 12	No additional treatment (*n* = 48)	13.4%	29.0%	–	*p* = 0.028
[62]	Dydrogesterone 20 mg/day (*n* = 175) until gestation Week 20	Placebo (*n* = 173)	6.9%	16.8%	–	*p* = 0.004
			**Rate of PL**			
**Meta-Analysis**	**Dataset**	**Main Result**	**Progestogen**	**Control**	**RR/OR** (**95% CI**)	***p*** **Value**
[82]	Dydrogesterone vs. placebo or conservative treatment (standard care) Three studies (*n* = 509)	Significant reduction in the miscarriage rate with dydrogesterone	10.5%	23.5%	OR: 0.29 (0.13–0.65)	NA ^a^
[42]	Progestogens vs. placebo or no treatment Ten studies (*n* = 1586); dydrogesterone vs. placebo or no treatment, three studies (*n* = 277)	Lower risk of miscarriage with progestogen treatment	–	–	RR: 0.72 (0.53–0.97)	*p* = 0.03
[2]	Progestogens vs. placebo or no treatment Ten studies (*n* = 1684); dydrogesterone vs. placebo or no treatment, three studies (*n* = 518)	Numerical decrease in the miscarriage rate with progestogen treatment (not significant)	20.1%	27.5%	RR: 0.73 (0.54–1.00) ^b^	*p* = 0.10

^a^ *p* value not stated; ^b^ two of the three studies using dydrogesterone showed a clear reduction in the risk of PL with progestogen treatment [62,81]. BID, twice daily; CI, confidence interval; NA, not applicable; OR, odds ratio; PL, pregnancy loss; RCT, randomized clinical trial; RR, risk ratio.

## Data Availability

Not applicable.
